# Does Doxycycline work in synergy with cisplatin and oxaliplatin in colorectal cancer?

**DOI:** 10.1186/1477-7819-7-2

**Published:** 2009-01-06

**Authors:** Jayesh Sagar, Kevin Sales, Sas Dijk, JanWillem Taanman, Alexander Seifalian, Mark Winslet

**Affiliations:** 1 Division of Surgery and Interventional Science, University College London, Gower Street, London, WC1E 6BT, UK; 2 Academic Department of Surgery, Royal Free & University College Medical School, Pond Street, London, NW3 2QG, UK; 3 Department of Clinical Neuroscience, Royal Free & University College Medical School, Pond Street, London, NW3 2QG, UK

## Abstract

**Background:**

In recent years, apart from antibacterial properties, doxycycline is reported to have cytotoxic and anti-proliferative actions in various cancers including colorectal cancer. Colorectal cancer constitutes one of the most common cancers in the western population. Apart from surgery, chemotherapy plays crucial role in the treatment of colorectal cancer. Cisplatin and oxaliplatin are most commonly used platinum compounds for the cancer chemotherapy. This study has looked for any impact of doxycycline on the cytotoxic effects of platinum compounds in colorectal cancer including its mechanisms of actions.

**Methods:**

HT 29 colorectal cancer cells were used for this study. These cells were treated with cisplatin and oxaliplatin with or without doxycycline treatment. The caspase 3 gene expression was quantitated by gel electrophoresis and qualitated by real time polymerase chain reactions. The caspase 3 activity was assessed in HT 29 cells with fluorescence kit.

**Results:**

The results revealed increased caspase 3 gene expressions and activities in HT 29 cells treated with cisplatin, oxaliplatin and doxycycline; however the combination of doxycycline with cisplatin and oxaliplatin did not report increased caspase 3 gene expressions and activity compared to cisplatin and oxaliplatin alone.

**Conclusion:**

We concluded that doxycycline has role in apoptosis induction in the colorectal cancer. However, it did not show any synergy with platinum compounds in the colorectal cancer cells. This study also pointed towards possible caspase-independent actions of doxycycline with cisplatin and oxaliplatin. However, further work is required to underpin the mechanisms of actions of doxycycline.

## Background

Tetracyclines (TCNs) have been used in clinical practice as antibiotics in various bacterial, mycoplasma, chlamydiae, rickettsiae and protozoan infections since more than 5 decades [1]. Their main mechanism of action involves inhibition of protein synthesis by restricting binding of aminoacyl t-RNA to 30S ribosomes [2]. Tetracyclines are believed to interfere the mitochondrial protein synthesis that let to the discovery of other effects, independent of their antimicrobial actions [3]. Recently, the renewed interest in the study of tetracyclines has evolved due to their ability to inhibit matrix metalloproteinase (MMPs) in various cancers such as prostate [4], melanoma [5], osteosarcoma [6], breast [7], leukaemia [8] and colorectal cancers [9]. Some of TCNs have been shown to work as apoptotic inducers [10]. Despite TCNs' emerging role as anti-invasive agents, their role in combination with other agents and the precise mechanisms are yet to be defined.

Apoptosis is the mechanism by which chemotherapeutic agents induce cancer cell death [11]. There are mainly two mechanisms/pathways of apoptosis, called intrinsic and extrinsic pathways. Caspases, the proteolyic enzymes, cysteine proteases, play essential role in execution of apoptosis [12]. Caspase 3 is one of the executioner caspases involved in both, the extrinsic and intrinsic pathways of apoptosis. Colorectal cancer is the third most common cancer in males and second most common cancer in females. It accounts for about 16,000 deaths per year in UK. Apart from surgery and radiotherapy, chemotherapy also plays significant role in the treatment of colorectal cancer. Cisplatin and oxaliplatin are two of most commonly used chemotherapeutic agents in the cancer chemotherapy of various cancers. However the problems of recurrence of disease and metastasis are not uncommon with these platinum compounds regimens. The advantages of doxycycline include long duration of actions and feasibility of oral administration. It has shown to inhibit cell proliferation and invasion and to induce apoptosis in colorectal cancer cell lines [13,14]. In this study we investigated whether doxycycline works in synergy with cisplatin and oxaliplatin or not in HT 29 colorectal cancer cell line.

## Methods

### Cell line

The human colorectal cancer cell line, HT 29 (purchased from ECACC) was used in experiments. The HT 29 cells were maintained in McCoy 5A media supplemented with 10% fetal bovine serum, penicillin (50 units/ml) and streptomycin (50 units/ml) at 37°C in a humidified atmosphere with 95% air and 5% CO_2_.

### Chemical reagents

Cisplatin (Bristol-Myers Squibb, UK), oxaliplatin (Sanofi-Synthelabo, UK) and doxycycline (Alpharma, UK) were used in these experiments. 50 μM of cisplatin and oxaliplatin were used (LD_50 _obtained from our preliminary study). Doxycycline was used in 10 μg/ml dose (dose obtained from our preliminary data from the dose response study of doxycycline).

### Reverse transcription and polymerase chain reaction

2 × 10^4 ^cells/ml HT 29 cells were plated in 6 well plates for 24 hours for cellular attachments followed by treatment with 50 μM of cisplatin or oxaliplatin with or without 10 μg/ml of doxycycline. After 1, 4 and 24 hours of treatment the cells were trypsinized and cell pellets were used for RNA extraction with RNeasy minikit (Qiagen, UK) according to manufacturer's instructions. The extracted RNA was used for reverse transcription and polymerase chain reaction (RT-PCR) for GAPDH (as control) and caspase 3 gene expressions with One-Step RT-PCR kit (Qiagen, UK) according to manufacturer's instructions. The primers used are – GAPDH Forward – 5'-AACTTTGGCATTGTGGAAGG-3' Reverse 5'-GGAGACAACCTGGTCCTCAG-3' and Caspase-3 Forward -5'-TGTCATCTCGCTCTGGTACG-3' Reverse -5'-AAATGACCCCTTCATCACCA-3'

The experiments were repeated three times. In all the experiments, doxycycline refers to the doxycycline treatment alone for 24 hours.

### Gel electrophoresis

The RT-PCR products were resolved in 2% agarose gel and electrophoresis was performed at 80 mV and 400 mA for 60 minutes. The gel stained with ethidium bromide was visualised under ultraviolet light and band width was measured by Synogene software programme. The experiments were repeated for three times independently.

### Real time polymerase chain reaction

The HT 29 cells were treated as per reverse transcription and the cells were trypsinized and cell pellets were used for RNA extraction with RNeasy minikit (Qiagen, UK) according to manufacturer's instructions. The caspase 3 and β actin (as standard) genes expression was determined using two steps. The first step of reverse transcription was performed with the Omniscript RT Kit (Qiagen, UK) according to manufacturer's instructions and the real time PCR was performed with Roche Molecular Lightcycler, UK. The experiment was repeated three times independently.

### Caspase 3 activity

2 × 10^4 ^cells/ml HT 29 cells were plated in 6 well plates for 24 hours for cellular attachments followed by treatment with 50 μM of cisplatin or oxaliplatin with or without 10 μg/ml of doxycycline. After 1, 4 and 24 hours of treatment, the cell pellets were collected through trypsinisation and caspase 3 activity was determined with Fluorometric Caspase 3 activity assay kit (R&D Systems, UK) according to manufacturer's instructions. The experiment was repeated three times independently.

### Statistical analysis

The t test was performed with Microsoft Excel, Microsoft Office 2003 for statistical analysis on the Microsoft XP system. The p value of < 0.05 was considered as statistical significant.

## Results

### RT PCR and gel electrophoresis

**Figure 1:**
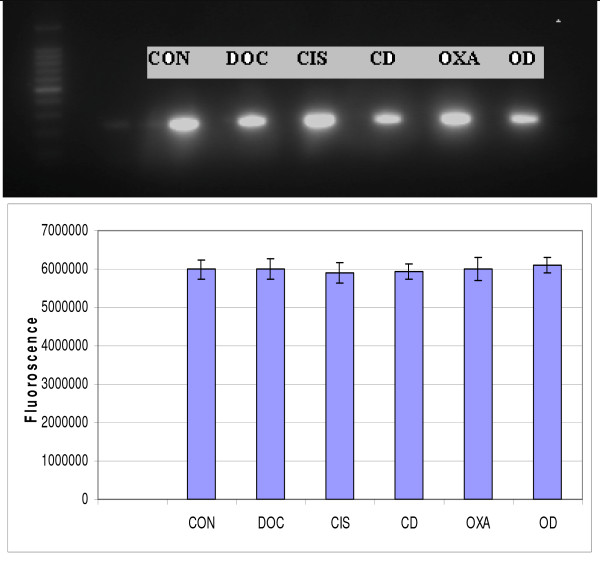
**Gel electrophoresis of GAPDH amplicons**. The upper part reveals qualitative assessment of GAPDH gene expression while lower part shows quantitative assessment in different cell samples. (CON = Control, DOC = Doxycycline, CIS = Cisplatin, CD = Cisplatin and Doxycycline, OXA = Oxaliplatin, OD = Oxaliplatin and Doxycycline). The data are depicted as means of three experiments ± standard deviation.

**Figure 2:**
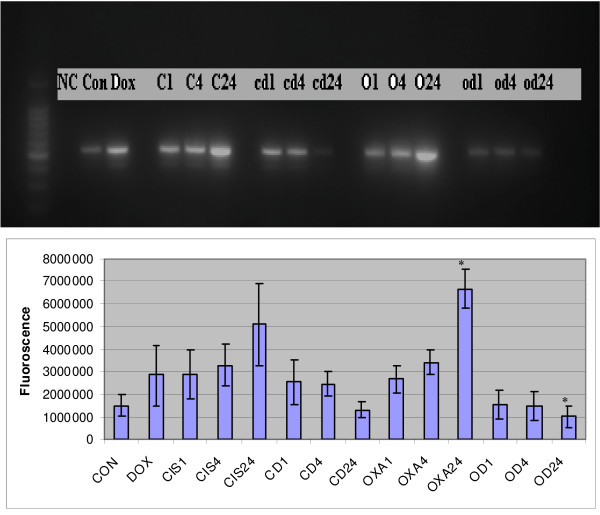
**Gel electrophoresis of Caspase 3 RT PCR products in HT 29 cells treated with cisplatin or oxaliplatin with or without doxycycline**. The upper part reveals qualitative assessment of caspase 3 gene expression while lower part shows quantitative assessment in different cell samples. (NC = Negative control, Con = CON = Positive control, Dox = DOX = Doxycycline treatment for 24 hours, C(CIS)1; C4; C24 = Cisplatin treatment for 1, 4 and 24 hours respectively, cd(CD)1; cd4; cd24 = Cisplatin and Doxycycline treatment for 1, 4 and 24 hours respectively, O(OXA)1; O4; O24 = Oxaliplatin treatment for 1, 4 and 24 hours respectively, od(OD)1; od4; 0d24 = Oxaliplatin and Doxycycline treatment for 1, 4 and 24 hours respectively, * – Statistically significant difference). The data are depicted as means of three experiments ± standard deviation.

The gel electrophoresis revealed presence of GAPDH amplicons in all the cell samples including control suggesting equal loading as an initial control (figure 1). It also showed increased caspase 3 transcripts in HT 29 cells treated with doxycycline compared to control (p = 0.21). The caspase 3 transcripts level increased in time dependent manner in HT 29 cells treated with cisplatin and oxaliplatin alone. However its level decreased in time dependent manner in HT 29 cells treated with combinations of cisplatin or oxaliplatin and doxycycline (p = 0.066 and p = 0.001 respectively) (figure 2).

### Real time PCR for Caspase 3 gene expression

**Figure 3:**
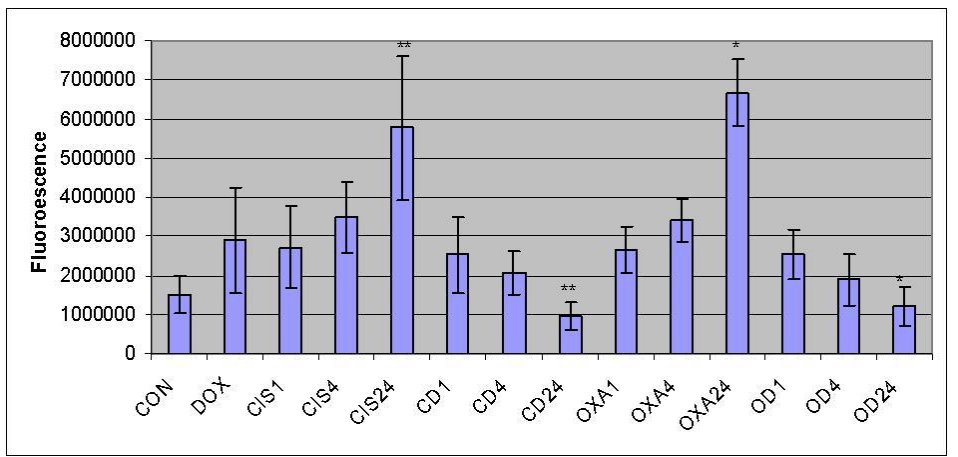
**Quantitative presentation of the real-time PCR of caspase 3 gene expression in HT 29 cells**. The HT 29 colorectal cancer cells were treated with cisplatin and oxaliplatin for 24 hours with or without doxycycline. The caspase 3 gene expression was measured in 2 steps; reverse transcription and real time PCR. (CON = Control, Dox = DOX = Doxycycline treatment for 24 hours, CIS1; 4; 24 = Cisplatin treatment for 1, 4 and 24 hours respectively, CD 1; 4; 24 = Cisplatin and Doxycycline treatment for 1, 4 and 24 hours respectively, OXA 1; 4; 24 = Oxaliplatin treatment for 1, 4 and 24 hours respectively, OD 1; 4; 24 = Oxaliplatin and Doxycycline treatment for 1, 4 and 24 hours respectively, **, * – Statistically significant difference). The data are depicted as means of three experiments ± standard deviation.

The two step real time PCR results revealed that the caspase 3 transcripts increased in the time dependent manner in the HT 29 colorectal cancer cells treated with cisplatin and oxaliplatin but caspase 3 transcripts levels decreased in the time dependent manner in the cells treated with cisplatin and oxaliplatin with doxycycline (p = 0.01 and p = 0.005) (figure 3).

### Caspase 3 activity

Caspase 3 activity studies reported time dependent increase in the activity levels in HT 29 cells treated with cisplatin and oxaliplatin. Doxycycline also showed increased caspase 3 activity compared to control (p = 0.034) (additional file 1). However, results did not reveal much increase in caspase 3 activity levels in cells treated with cisplatin and oxaliplatin in combination with doxycycline (p = 0.001 and p = 0.039 respectively). These findings corresponded with the findings obtained in caspase 3 gene expressions.

## Discussion

Colorectal cancer constitutes the third most common cancer and the second leading cause of cancer deaths in UK. About one in 20 men develops colorectal cancer in their lifetime (Cancer Research UK 2005, Cancer Stats). Apart from surgical intervention and radiotherapy, chemotherapy plays crucial role in the management of colorectal cancers. The main reasons for the mortality from colorectal cancers are metastases or recurrence of the disease. Recently, doxycycline has been reported to have cytotoxic and anti-proliferative actions on various cancer cells [3,4,14-18]; however, the exact mechanisms of apoptosis are not well understood. Doxycycline has also been reported to have anti-invasive properties in cancer cells[3,13]. To some extent, these effects of doxycycline may be co-related with its actions on mitochondria, where it induces reduction in protein synthesis through inhibitory effects on oxidative phosphorylation. However, the definite mechanisms of doxycycline are yet to be reported. According to literature, there has been only one study which looked into the combination effects of doxycycline with other agent [13]. This study answered whether doxycycline has any synergistic effects with other cytotoxic agents, the platinum compounds; cisplatin and oxaliplatin exploring the mechanisms of action on colorectal cancer cells at the same time.

Caspases are the cysteine proteases which are crucial for the process of apoptosis. Caspase 3 is one of the effector caspases in the apoptosis cascade. It is activated from procaspase 3 through intrinsic as well as extrinsic pathways and results in cell death. Increased caspase 3 gene expression or activity is considered as one of the hall mark of the apoptosis induction. We studied caspase 3 gene expression and activity levels to assess any synergic/additive impact of doxycycline on the effects of cisplatin and oxaliplatin. From the review of literature, it was concluded to use sub lethal dose of 10 μg/ml of doxycycline in these experiments, a level that has been reported in the gingival fluid following a systemic dose of doxycycline, and has been shown to arrest G_0_/G_1_cell cycle in HT 29 cells. The caspase 3 gene expression revealed a time dependent increase of expression in the cells treated with cisplatin and oxaliplatin but there was time dependent decrease in caspase 3 transcripts in the cells treated with combination of drugs (figure 2) This difference was statistically significant in the HT 29 cells treated with oxaliplatin compared to combination of oxaliplatin and doxycycline (figure 2). These findings were confirmed by real time PCR studies which showed statistically significant difference in the caspase 3 gene expression in HT 29 cells treated with cisplatin or oxaliplatin in comparison with their combination treatment with doxycycline respectively (figure 3).

**Figure 4:**
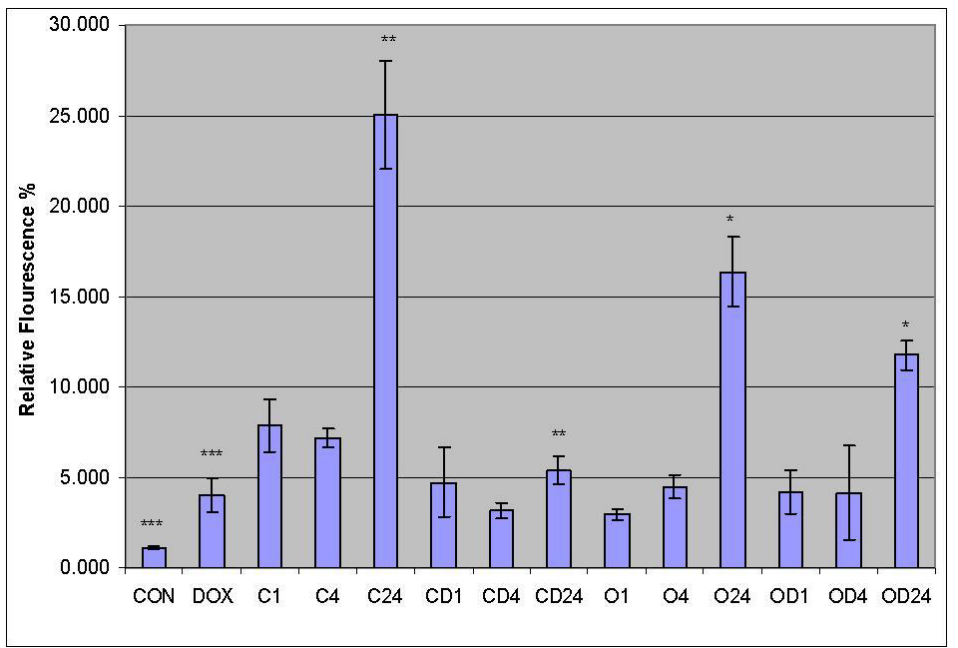
**Quantitative presentation of caspase 3 activity in HT 29 cells**. The HT 29 colorectal cancer cells were treated with cisplatin or oxaliplatin with or without doxycycline and caspase 3 activity was measured. (Con = CON = Positive control, Dox = DOX = Doxycycline treatment for 24 hours, C1; C4; C24 = Cisplatin treatment for 1, 4 and 24 hours respectively, CD1; CD4; CD24 = Cisplatin and Doxycycline treatment for 1, 4 and 24 hours respectively, O1; O4; O24 = Oxaliplatin treatment for 1, 4 and 24 hours respectively, OD1; OD4; 0D24 = Oxaliplatin and Doxycycline treatment for 1, 4 and 24 hours respectively. *, **, *** – Statically significant difference). The data are depicted as means of three experiments ± standard deviation.

As mentioned earlier, Onoda et al were the only one who reported the impact of combination of doxycycline and cyclooxygenase-2 inhibitor in colorectal cancer cells [13]. Although they showed increased caspase 3 activity following doxycycline treatment, they did not study caspase 3 activity following the combination treatment of doxycycline and cyclooxygenase-2 inhibitor. However, they reported inhibition of cell proliferation and attenuated matrix metalloproteinase expression following combination treatment. Our study showed similar results in the form of increased caspase 3 activity following doxycycline treatment in HT 29 cells, but showed decreased caspase 3 activity in cells treated with combination treatment. Therefore, to verify our findings, the caspase 3 activity assay was performed that reported statistically significant difference in the caspase 3 activity in HT 29 cells treated with cisplatin and oxaliplatin alone in comparison with combination of cisplatin/oxaliplatin and doxycycline (figure 4). Although the caspase activity was significantly low in the cells treated with combination therapy compared to platinum compounds, doxycycline on its own showed statically significant caspase 3 activity compared to control (figure 4).

To clarify the impact of caspase 3 mediated apoptosis in the cytotoxic effects of doxycycline alone as well as in combination with platinum compounds, a general cell viability assay was considered appropriate. In our preliminary study, Alamar blue assay was used for cell viability study. This cytotoxicity assay revealed statistically no difference in the cytotoxicity of platinum compounds compared to the combination of doxycycline with platinum compounds (additional files 1 &2). Thus combination of doxycycline with platinum compounds were reported to be no superior or synergistic to effects of platinum compounds in the colorectal cancer cells which contradict the findings of Onoda et al [13].

These findings have raised new questions about the role of doxycycline. It is possible that doxycycline may not have any synergistic actions with platinum compounds and may work on its own as cytotoxic, anti-proliferating or anti-invasive agent or doxycycline may have caspase-independent actions, especially in combination with platinum compounds. The advantages of doxycycline over other tetracyclines are its longer duration of action and comparatively less toxicity and thus may be considered as a potential candidate in future for cancer chemotherapy on its own or in combination with other agents, however to clarify the exact role of doxycycline alone as well as in combination with other agents and to underpin its mechanisms of actions, further study needs to be done.

The other key finding of this study was the similar findings obtained by caspase 3 gene expression and caspase 3 activity studies. We suggested similar efficacy of caspase 3 gene expression study and caspase 3 activity studies. The caspase 3 gene expression assay is expensive and more complex procedure compared to caspase 3 activity assays and thus we recommend the caspase 3 activity assay rather than caspase 3 gene expression study as cost effective procedure for future studies. We concluded that doxycycline may be useful as an apoptotic agent; however its role in combination with other chemotherapeutic agents needs to be clarified by further studies. These findings have opened the new avenues of further research.

## Conclusion

We conclude that doxycycline has role in apoptosis induction in colorectal cancer cells, possibly via caspase 3 activation, however in combination with platinum compounds; it did not show any synergic or additive effects. This study also suggests possible role of caspase-independent mechanisms of doxycycline when combined with platinum compounds. Further study needs to be carried out to outline the exact mechanisms of actions of doxycycline alone as well as in combination. We also recommend use of caspase 3 activity assay compared to more complex and more expensive caspase 3 gene expression study for caspase studies.

## Competing interests

The authors declare that they have no competing interests.

## Authors' contributions

JS was the main author and contributed in the all aspects of this work including preparation of manuscript. KS contributed in cell culture work. SD contributed in caspase gene expression studies. JWT contributed in caspase activity studies. MW contributed in the form of supervision and guidance and helped in preparation of manuscript. AS contributed to the preparation of the manuscript. All authors have read and agreed for the submission of this manuscript.

## Authors' note

The methodology for Alamar blue assay has not been include in the methods section as it was part of the preliminary experiments, however if required please do not hesitate to contact the author.

## Supplementary Material

Additional file 1 **Doxycyclin sup 1**. Microsoft office file showing Cytotoxicity of cisplatin with or without doxycycline in HT 29 cells. The HT 29 cells were treated with different concentrations of cisplatin (0 – 1000 M) with or without 10 μg/ml of doxycycline for 24 hours and the cytotoxicity assay was performed with Alamar blue assay according to the manufacturer's instructions.(● – cisplatin, ■ – cisplatin and doxycycline,). Data are depicted as means of three experiments ± standard deviation. (*p *= 0.84). Click here for file

Additional file 2 **Doxycyclin sup 2**. Microsoft office file showing Cytotoxicity of oxaliplatin with or without doxycycline in HT 29 cells. The HT 29 cells were treated with different concentrations of oxaliplatin (0 – 1000 M) with or without 10 μg/ml of doxycycline for 24 hours and the cytotoxicity assay was performed with Alamar blue assay according to the manufacturer's instructions.(● – oxaliplatin, ■ – oxaliplatin and doxycycline,). Data are depicted as means of three experiments ± standard deviation. (*p *= 0.95). Click here for file
