# Supplementary material for: Does Doxycycline work in synergy with cisplatin and oxaliplatin in colorectal cancer?
Source: World J Surg Oncol. 2009 Jan 6;7:2. doi: 10.1186/1477-7819-7-2 (PMC2628910; doi:10.1186/1477-7819-7-2)
Supplement: Additional file 1 — Doxycyclin sup 1. Microsoft office file showing Cytotoxicity of cisplatin with or without doxycycline in HT 29 cells. The HT 29 cells were treated with different concentrations of cisplatin (0 – 1000 M) with or without 10 μg/ml of doxycycline for 24 hours and the cytotoxicity assay was performed with Alamar blue assay according to the manufacturer's instructions.(● – cisplatin, ■ – cisplatin and doxycycline,). Data are depicted as means of three experiments ± standard deviation. (p = 0.84). [file 1477-7819-7-2-S1.doc]

0

10

20

30

40

50

60

70

80

90

100

0

1

2

3

**Log dose cisplatin (Molar)**

**% Control of relative fluorescence**
